# Alveolar Echinococcosis in the Early 2020s: A Systematic Review

**DOI:** 10.3390/pathogens15020132

**Published:** 2026-01-26

**Authors:** Bogdan-Florin Ciomaga, Mihai-Octav Hogea, Andrei-Alexandru Muntean, Mădălina-Maria Muntean, Mircea Ioan Popa, Gabriela Loredana Popa

**Affiliations:** 1 Department of Microbiology II, Carol Davila University of Medicine and Pharmacy, 020021 Bucharest, Romania; bogdan-florin.ciomaga@drd.umfcd.ro (B.-F.C.); octav.hogea@umfcd.ro (M.-O.H.); alexandru.muntean@umfcd.ro (A.-A.M.); 2 Department of Microbiology, Cantacuzino National Military Medical Institute for Research and Development, 050096 Bucharest, Romania; 3 Department of Parasitology, Carol Davila University of Medicine and Pharmacy, 020021 Bucharest, Romania; madalina.muntean@umfcd.ro (M.-M.M.); gabriela.popa@umfcd.ro (G.L.P.); 4 Parasitic Disease Department, Colentina Clinical Hospital, 020125 Bucharest, Romania

**Keywords:** alveolar echinococcosis, epidemiology, prevention and control, systematic review

## Abstract

Background: Alveolar echinococcosis (AE) is a neglected parasitic disease caused by *Echinococcus multilocularis* that is difficult to diagnose and treat. Methods: This systematic review has gathered articles presenting original data from the past 5 years, from January 2020 to December 2025, with epidemiological data (incidence, prevalence), treatment options, case reports, and other findings relevant to the prevention and control of this disease, representing the inclusion criteria of this study. Three medical databases were searched for the study: PubMed, Web of Science, and ScienceDirect. To improve our understanding of the available data, no spatial or temporal restrictions were imposed on the study’s duration or follow-up period. Results: A total of 248 articles are included in this review, which describe atypical sites and complications owing to *Echinococcus multilocularis* infection, the heterogeneity of epidemiological studies in different endemic and non-endemic regions, diagnosis techniques based on imaging, histopathology, and molecular techniques, as well as surgical and non-surgical treatment options (and lack thereof regarding the latter). Conclusions: Although advances have been made in the diagnosis, management, and treatment of AE, challenges remain, particularly with regard to misdiagnosis, delayed diagnosis, and limited antiparasitic therapy.

## 1. Introduction

Alveolar echinococcosis (AE) is a neglected zoonotic disease caused by *Echinococcus multilocularis* [1]. The adult platyhelminth lives in the intestines of vulpines. However, it has also been found in other predatory animals, including canids and felids [2]. This also explains the geographic distribution, owing to its sylvatic lifecycle [1]. Furthermore, AE is reported to occur only in the Northern Hemisphere, owing to its temperate climate [1,2], although recent reports indicate that the parasite can thrive in Arctic and subarctic environments, as well as in high-altitude grasslands and urban areas [3]. The eggs of the adult usually reach the intermediate host (ruminants and humans) via fecal–oral transmission, where they develop into the metacestode stage [1,2]. In the usual lifecycle, the intermediate host is then hunted and consumed by predators, allowing the metacestode to enter the definitive host and develop into an adult tapeworm within the intestines [1].

In humans, the initial metacestode infection typically develops in the liver, where the infection can persist asymptomatically for years [1]. The disease is usually discovered incidentally during routine imaging. However, it may also be uncovered when the parasitic lesion enlarges to the point of causing symptoms (cholangitis, thrombosis, tissue necrosis and subsequent bacterial superinfection, and, in some cases, Budd–Chiari Syndrome, represented by the triad of ascites, hepatomegaly, and abdominal pain) [1]. In later stages of infection, the parasitic lesion may spread through the hematogenous dissemination of protoscoleces to other organs, with the most common secondary sites of infection being the lung and brain [1,4].

There is ongoing discussion about the optimal imaging technique for diagnosis, with ultrasound (US), computed tomography (CT), and magnetic resonance imaging (MRI) each presenting advantages and disadvantages, and the tumor-like or hemangioma-like appearances of the metacestode posing numerous challenges [1,5]. Regardless of the imaging method, it is usually challenging to confirm *E. multilocularis* infection without confirmatory diagnostic methods, and thus, serologic testing and biopsy are warranted as confirmatory tests [1,5].

Due to the variation in the lesion’s location, its particular interaction with the surrounding tissue, and its presentation, *E. multilocularis* has proven difficult to classify, especially compared to the relatively straightforward classification methods of *E. granulosus.* In response, the World Health Organization Informal Working Group on Echinococcosis (WHO-IGWE) has proposed a classification system that mirrors the TNM classification in oncology—the PNM classification (P—parasitic lesion; N—neighboring organs; and M—metastases) [6]. Although less widely used than the cystic echinococcosis (CE) classification method for *E. granulosus*, it has been more frequently cited in recent publications.

As alveolar echinococcosis remains classified as a neglected disease, the need for further studies is underscored. This review aims to synthesize recent findings on the epidemiology, clinical presentation, and treatment of human alveolar echinococcosis and to correlate these data with prior information.

## 2. Materials and Methods

As a systematic review, the authors state that our work adheres to the PRISMA reporting guidelines and that our review protocol was not registered [7].

Three medical databases were searched for the study: PubMed, Web of Science, and ScienceDirect. Articles published in English from January 2020 to December 2025 were included. To improve our understanding of the available data, no spatial or temporal restrictions were imposed on the study’s duration or follow-up period.

During data collection, the Medical Subject Headings (MeSH) technique was employed, and parallel strategies employing identical keywords were applied across the available databases. Studies were identified based on the content of their titles and abstracts, using keywords such as “*Echinococcus multilocularis*”, “Alevolar Echinococcosis”, “prevalence”, “incidence”, “treatment”, “prevention”, and “clinical”, both individually and with Boolean operators AND and OR. Inclusion criteria included articles that offered original clinical findings, epidemiological data, treatment options, case reports, and other findings relevant to the prevention and control of this disease. Full-text analysis was performed on the articles that passed the initial selection, and the articles were sorted by content.

Exclusion criteria included articles written in a language other than English, articles that were not relevant to the subject matter of this review (ex., Alveolar echinococcosis was not the topic addressed, there was no human addressability, etc.), other reviews, articles that only discussed non-human hosts, articles that only discussed *Echinococcus granulosus*, duplicates, retracted articles, and errata.

Two authors independently screened each database. They evaluated each article’s eligibility individually. After screening, articles with unclear eligibility status were assessed concurrently by both authors. If a decision could not be made, the entire team would be involved in determining eligibility. No automation tools were used in the assessment process.

Once the data collection process was completed, articles were organized by content into the following categories, presented in Section 3: Pathology and Lifecycle, Epidemiology, Diagnosis, and Treatment.

## 3. Results

Following the methodology, the first queries yielded 3083 results. After duplicates were removed (*n* = 91), the remaining studies were screened (*n* = 2992). After excluding manuscripts based on title and abstract (*n* = 835), the remaining 2157 manuscripts were further assessed for eligibility. Of those, 491 were reviews; 455 discussed only non-human hosts; 399 addressed *Echinococcus granulosus*; 338 were irrelevant to the topic of the evaluation; 210 were not written in English; and 9, 6, and 1 were erratum papers, comment papers, and retracted papers, respectively, and were thus excluded from the study.

After screening, 248 studies were deemed eligible for inclusion in the current work.

**Figure 1:**
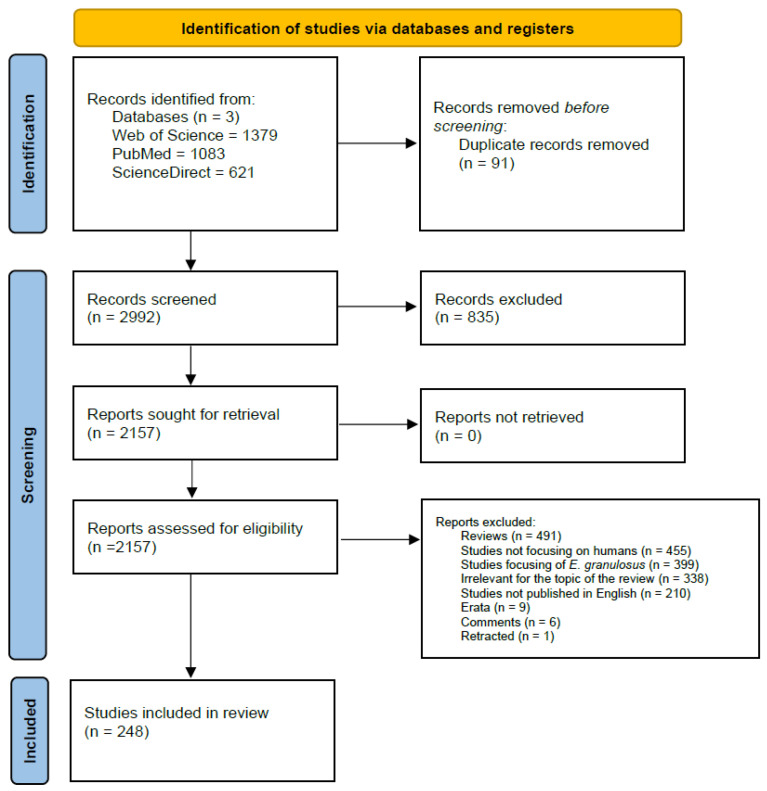
Flowchart of the process of selection of articles.

For a detailed overview of the process, please refer to Figure 1 and Supplementary Tables S1, S2 and S3.

### 3.1. Pathology and Lifecycle

#### 3.1.1. Primary Lesion

Although the classic pathology of *Echinococcus multilocularis* is a single primary hepatic lesion, and the vast majority of cases do seem to adhere to this pattern, other locations for the primary lesion have also been described. The larva’s primary location has been reported in the brain [8,9], lung [8,10], bone [11,12,13], skin [14], the parotid gland [15], and the epididymis [16]. In all cases, *E. multilocularis* was not initially considered, and the diagnosis of AE was made only after subsequent testing, thereby delaying appropriate care. A “primary” lesion of sorts can also occur at atypical sites in the context of transplantation; cases have been described in which the transplanted organ was infected with *E. multilocularis* prior to transplantation, resulting in a patient presentation of an extrahepatic AE infection [17].

A case of *E. multilocularis* without a primary hepatic lesion has also been described by Joyce et al., in which the parasitic growth was discovered in the left kidney [18]. Atypically, in this situation, the patient was immunocompetent; a possible explanation was that the presence of a porto-systemic shunt may have interfered with the parasite’s path towards the liver. The primary lesion can also occur in the liver following transplantation with infected tissue [19,20].

It should also be noted that, although extremely rare, infection with *E. granulosus* does not preclude coinfection with *E. multilocularis*, as cases have been reported in which both pathogens infected the same person, adding confusion and further complicating diagnosis and treatment [11,21,22].

#### 3.1.2. Secondary Lesion

As noted in the introduction, beyond the primary lesion, *E. multilocularis* protoscoleces can disseminate throughout the body, as in metastatic cancer, either hematogenously or by invading neighboring organs. In the articles included in this review, we have found secondary lesions being described in the following organs/locations: lung [8,23,24,25,26,27], adrenal glands [8,26,28], brain [8,23,26,27,29], peritoneum [8,30], spleen [8], diaphragm [8,23,31], kidney [8], vertebrae [8], chest wall [32], heart [8,33], mediastinum [8], muscle [8], pancreas [8], and also lymph nodes [34,35].

Secondary lesions represent another hurdle in providing adequate treatment for the patient, as they are often misdiagnosed as oncological metastases; this is particularly disastrous in the case of brain lesions, as the treatment of brain metastases (radiotherapy) does not affect *E. multilocularis* lesions [36].

#### 3.1.3. Complications

One of the most commonly seen complications of AE infection is Budd–Chiari syndrome, wherein the lesion causes blockage of blood flow in the liver [37]. This symptomatology often requires surgery to alleviate the obstruction, with either autotransplantation or donation of liver tissue [37,38].

Bacterial superinfection is another potential complication; the anaerobic conditions created by the parasitic lesion can foster bacterial growth, such as Clostridium perfringens, thereby complicating patient management [39]. Coinfection with tuberculosis was also reported [16], as was a case of *E. multilocularis* osteomyelitis with superinfection by *Corynebacterium tuberculostearicum* [13].

Depending on the duration of the infection, treatment options—specifically surgical interventions—may be ineffective at times, worsening the patient’s condition. Pielok et al. describe how such an endeavor resulted in portal hypertension, splenomegaly, and symptoms of liver cirrhosis [40].

A higher percentage of patients with *E. multilocularis* infection have also been found to harbor *Blastocystis* spp., a single-celled parasite, compared with healthy individuals [41].

### 3.2. Epidemiology

Given the disease’s neglected status and the availability of epidemiological data, the evidence is considered weak. With this in mind, some countries have conducted epidemiological surveillance of AE infection. Asian countries such as China [27,42,43,44,45,46,47,48,49,50,51,52,53,54,55,56,57,58,59], India [60,61,62], Kyrgyzstan [63,64,65], and Pakistan [66]; European countries [67] such as Slovakia [68], Switzerland [65,69,70], Germany [71,72], France [73], and Hungary [74,75]; and North American countries such as the United States of America [76] and Canada [77] tend to offer the most comprehensive epidemiological investigations. Additionally, countries such as Chile develop documents to properly assess the epidemiological considerations surrounding AE [78]. Furthermore, non-endemic regions worldwide, such as Norway [79], have begun to provide insights into how they address alveolar echinococcosis. One critical aspect observed is how each research team presented the data; heterogeneity is partially explained by experience with the pathology in an epidemiological context: Norway is a non-endemic region, whereas China is considered endemic, as evidenced by the number of studies published during the chosen time frame.

Molecular epidemiology has become increasingly important for defining local strains, with studies being performed in France [80,81,82], Belgium [83], Turkey [84,85], China [86,87,88,89,90], Pakistan [91], and Kyrgyzstan [64].

Current studies aim to better understand the formal epidemiological setting of the disease, defining metrics such as incidence [53,72,92,93] and prevalence, both in the human host as well as definitive hosts such as foxes, coyotes, and dogs [25,49,50,94,95,96,97], and then to focus on identifying risk factors, and thus, vulnerable populations, so that potential control and prevention interventions can be as effective as possible [98,99,100,101,102,103].

Furthermore, cases of autochthonous disease have started to emerge in the literature from countries such as Italy [104], Croatia [105], Poland [106], Serbia [107], and Inner Mongolia [42,108].

Insights into rare conditions and their inter-relationships with alveolar echinococcosis have also begun to emerge. Associations with cystic echinococcosis with [109,110] or without [11] liver involvement, epilepsy [92], metastasis [35], heart–liver transplantation [33], and other solid organ transplantations [20], adrenal gland [28], and nerve infiltrations have been reported [111]. Rare forms of alveolar echinococcosis have also been documented, such as disseminated echinococcosis [18,112,113,114], cerebral alveolar echinococcosis [115,116], subcutaneous swelling [14], cardiac echinococcosis [117,118,119], vertebral osteomyelitis [120,121], intra- and intermuscular cyst [122], thyroid gland cyst [123], jaundice with segmental obstructive cholestasis [124], and cholangiocarcinoma-mimicking symptoms leading to liver transplant [125,126].

### 3.3. Diagnosis

#### 3.3.1. Imaging

Imaging remains one of the most important diagnostic methods for *Echinococcus multilocularis*; however, differential diagnosis often results in significant delays between investigations and definitive diagnosis, leading to most patients being diagnosed at advanced stages of disease [127]. Furthermore, the presence of multiple classification methods, such as the Ulm classification for CT [39,128], PNM classification [129,130], and Kodama-XUUB classification for MRI [131,132,133], can further complicate patient diagnosis and the establishment of prognosis and treatment options. Due to limitations in certain classification systems, alternative methods have also been implemented, such as a deep learning-assisted CT classification system [134,135].

Imaging techniques for diagnosis typically include CT, MRI, and ultrasound (US), all of which are used to create a comprehensive image of the lesion [132].

For CT, the most commonly used method is ^18^F-fluorodeoxyglucose [^18^F][FDG] positron-emission tomography with computed tomography (PET/CT) [129,136,137,138,139,140,141,142]. Its main advantage is the ability to detect differences between the microenvironment of healthy tissue and that of the parasitic lesion [143]. Recently, Ayituhongman et al. also described the use of ^11^C-acetylcholine (^11^C-CHO) PET/CT for the detection of cerebral AE, with greater accuracy compared to [^18^F][FDG]-PET/CT [29].

MRI is beneficial for extrahepatic lesions and is typically used either in conjunction with CT scans [8] or as a standalone imaging technique [144].

As US is the easiest and most readily available of the three imaging methods described, its use is particularly evident in follow-up investigations [144] and epidemiological surveys [54,95]. US can also differentiate between AE, CE, and hepatoblastoma [145]. It can also serve as a less invasive imaging modality for follow-up investigations compared with PET-CT. Nevertheless, correlation of serological markers with US data remains necessary [146,147].

One potential avenue for further research is the use of artificial intelligence (AI) and machine learning techniques to refine the diagnosis of alveolar echinococcosis. So far, researchers have developed convolutional neural network models to further improve diagnostic accuracy. One such example, tested at scale in China, focused on ultrasound differentiation of alveolar echinococcosis from other focal liver lesions and demonstrated results comparable to, or better than, those of senior radiologists [148]. Similarly, machine learning approaches have been used to differentiate cerebral alveolar echinococcosis from brain metastases [36]. Other perspectives include the use of epidemiological models to understand how human prevalence varies across host populations (e.g., coyotes) in urban settings, such as Calgary, Alberta (Canada) [149], and a geographical–meteorological indicator system that enables researchers to develop predictive models for alveolar echinococcosis [150]. Similar approaches could be applied for CT images [135] and other imaging techniques.

Although imaging alone can provide information about patient status, nomogram analysis can also be used for prognosis and risk assessment. Nomogram analysis has been detailed for the prognosis of cerebral metastasis [151] and lung metastasis [152], using information such as lesion size, presence of metastasis in other organs, cavitary lesions, and enhancement characteristics [152], or lesion size, eosinophil count, and inferior vena cava (IVC) invasion [153]. A CT-based predictive model has also been developed to estimate the odds of lymph node metastasis [154].

A recent development in imaging technology is fluorescence imaging in secondary near-infrared (NIR-II), wherein photoluminescent contrast agents are used to generate fluorescence once they have gathered within specific organs or structures. Additionally, nanoprobes which specifically target *Echinococcus multilocularis* structures in vivo can be used to identify parasitic lesions in early stages in which other imaging techniques may fail, though this technique, to our knowledge, has yet to be used in clinical settings [155].

It is critical for medical professionals to understand the limitations of these methods and always corroborate their imagistic findings with clinical data. AE’s initial symptoms can be considered nonspecific; thus, suspected diagnoses need further methods to confirm the suspicion. Failing to address other potential causes could lead to misdiagnosis [156].

#### 3.3.2. Serology

To support imaging findings and distinguish from oncological pathology, serology is also performed. Typically, serology involves detecting antibodies against *Echinococcus* spp. IgG through indirect hemagglutinin assay (IHA) [146,157,158], as well as antibodies against *E. multilocularis* vesicle fluid (EmVF) [65], antibodies against antigenic structures Em2 and Em18 (also known as Em2+) [20,146,159], recombinant Em18 (rEm18) [133,160], and Em95 [65], using specialized enzyme-linked immunosorbent assays (ELISAs). Em2+ and IgG are useful in epidemiology for screening and for differentiating between AE and CE patients [54,146,161]. At the same time, rEm18 and Em2+ correlated with IgG may also be helpful for clinical monitoring, with Em2+ being a significant predictor of remission [146,158].

*Echinococcus multilocularis*-specific IgE levels were also found to be relevant for monitoring disease progression, with rising titers predicting progression; in contrast, total IgE levels did not vary over time [158].

Although serological tests have very high specificity, sensitivity can be low, and cross-reactivity with CE is expected [65]. This can also lead clinicians to underestimate positive diagnostic findings, particularly when the titer is very close to the cutoff [162].

Antibodies released by AE patients can cross-react with *Echinococcus granulosus* hydatid fluid (EgHF); therefore, *E. multilocularis* infection should be considered when a patient presents with positive anti-EgHF results and imaging findings that are incompatible with CE [138].

Beyond formal diagnosis, anti-rEm18 antibody titers also have use in follow-up after resection, with low values compared to initial preoperative values indicating a lower risk of recurrence [163].

#### 3.3.3. Histopathology

Histopathological investigation of either biopsy samples or resection specimens can confirm the diagnosis when imaging or serology alone are insufficient; furthermore, it is particularly valuable in cases of misdiagnosis, as biopsy is often performed to obtain histopathological information regarding potentially oncological pathology [157,164,165].

Briefly, histopathological diagnosis is based on the presence of a laminated layer that stains positively with periodic acid–Schiff staining. This layer is difficult to visualize with the commonly used method of hematoxylin staining [166]. Diagnosis can also be achieved by immunohistochemistry using anti-Em2G11 antibodies that preferentially bind to the laminated layer of *E. multilocularis* [10,166] or by mAbEm18, which also assesses the viability of AE lesions [167]. A novel antigen, EmG3, which is present in both AE and CE, can be used similarly [10].

#### 3.3.4. Molecular Diagnosis

Amplification of *E. multilocularis* DNA by polymerase chain reaction (PCR) found in biopsy and resection tissue samples can also constitute a viable diagnosis method, with a real-time multiplex PCR kit being designed for several species, including *E. multilocularis*, *E. granulosus sensu stricto*, *E. ortleppi*, and *E. canadensis* [168], another real-time multiplex PCR kit focused on *E. multilocularis*, *E. granulosus sensu lato (s.l.)*, and *Taenia* spp. [169], and another focused on detecting *E. multilocularis* circulating free DNA, which was found to have significant value for screening [170]. However, PCR diagnosis has limitations; in one case, it was considered only retrospectively, with a second biopsy being chosen for histological diagnosis instead [164].

Metagenomic next-generation sequencing (mNGS) can also be used to identify *E. multilocularis* DNA in patient samples, including abdominal lesions [171], cerebrospinal fluid in cases of cerebral AE [26], and lung-puncture samples [172].

#### 3.3.5. Other Diagnostic Tools

Cluster of differentiation 155 (CD155), a protein involved in the immune response, may increase in response to vascular invasion by *E. multilocularis*; elevated levels of its soluble form (sCD155) may be used as a diagnostic tool to stage disease progression [173].

Long non-coding RNA chains [174] and metabolomic profiling [175] may also have diagnostic utility, particularly for early-stage *E. multilocularis* diagnosis, where misdiagnosis is common.

The use of machine learning algorithms combined with spectroscopy, specifically shifted excitation Raman difference spectroscopy, may be one avenue for further research. This is a non-invasive screening strategy with high diagnostic accuracy, including in the early stages of the infection [176,177].

### 3.4. Treatment

#### 3.4.1. Surgery

As with prior findings, surgery remains the main treatment option for AE [178]. Resection of the parasitic lesion while maintaining a safety margin from healthy tissue remains the standard of care, preferably with perioperative benzimidazole treatment [179]. Robotic-assisted surgery has been successfully performed for AE [180,181]. Where possible, classical hepatectomies are attempted, with more complex surgical interventions attempted when the parasitic lesion makes classical techniques impossible, as described further below.

Anatomic hepatectomies are reported to have a lower risk of postoperative complications than non-anatomic hepatectomies [182,183]. Furthermore, some studies point out that partial cystectomy was associated with a higher risk of morbidity and complications, such as biliary fistula, when compared to radical surgery [184]. Although classical open approaches are preferred, laparoscopy has also been used in some cases of AE, with favorable outcomes [185,186], making it potentially more successful than equivalent open surgery. However, further research is needed [187]. Clinicians may also consider the Child–Pugh and Albumin–Indocyanine Green Evaluation grading systems to estimate hepatectomy success and limit postoperative complications [188].

Vascular involvement is a cornerstone of AE management, with disease severity linked to vascular pathology and to the parasitic lesion’s invasion of major blood vessels [189].

Several advances have been made in the techniques used for the resection of the lesion from liver tissue, of which one of the most important is the practice of ex vivo liver resection and autotransplantation (ELRA), where a significant portion of the liver is extracted, lesion resection is performed, and then the remaining tissue is autotransplanted back into the patient [37,38,181,190,191,192,193,194,195,196,197]. This technique also allows for inferior vena cava (IVC) [198,199,200,201,202], arterial [203], and biliary [204] reconstruction in cases where the lesion has progressed towards those structures, greatly extending the range of clinical presentations that can be successfully treated through surgical methods, especially for end-stage infections. Generally, ELRA is indicated for patients with lesions extending towards the portal vein, hepatic veins, and/or arteries (P4 on the PNM scale), with or without neighboring organ invasion (N0 or N1) and metastases (M0 or M1) [190]. It is also indicated for patients suffering from Budd–Chiari syndrome [37].

The main drawbacks of ELRA include the risk of complications, such as blood loss, that increase surgical time [190,205]; the procedure’s complexity [205]; and postoperative complications, including liver failure or infection [206]. Even so, in many cases where traditional liver resection is insufficient, the procedure can be curative, provided it is performed promptly after diagnosis [190]. Furthermore, some complications, such as biliary complications, can be accounted for by predictive models [207].

In cases where there is too little healthy liver tissue left, another pre-resection option for the patient is hepatic lobe hyperplasia [208].

In the case of advanced AE patients, specifically with hepatorenal AE, one course of action taken could be simultaneous combined surgery as a curative treatment. Tulahong et al. reported that patients did not face relapse or other significant complications at 37 months after their respective interventions [209].

A newer, “remnant liver-first” strategy is represented by in vivo resection combined with autotransplantation (IRAT), in which healthy liver tissue is first extracted and prepared as an autograft, followed by resection of infected tissue and its autotransplantation [205]. As this is a newer method, the exact use case for this procedure has not yet been defined.

Another technique previously applied in oncological settings that may also be useful for *E. multilocularis* infection is ultrasound-assisted percutaneous microwave ablation, particularly in cases where the parasitic lesion is located in dangerous or difficult-to-access regions [86,186,210,211]. Although operative time and postoperative complications were significantly lower with ablation than with hepatectomy, recurrence rates were higher, and ablation alone is insufficient without associated albendazole therapy [186].

Beyond the resection of liver lesions, AE patients should also be evaluated for lung lesions, for which surgery is the most preferred method of treatment, where it is available; approaches usually involve thoracotomy or transdiaphragmatic or laparoscopic methods [212].

Even when surgery can no longer achieve a curative effect, there is a case for partial resection of the parasitic lesion to provide relief and improve the patient’s quality of life [213,214].

One promising avenue of research is the use of artificial intelligence to determine whether to undergo surgery. Methods have been developed to enable medical teams to receive assistance from machine learning software, with promising results [215].

#### 3.4.2. Transplant

Beyond autotransplantation, transplantation of healthy liver tissue from compatible donors can also assist in treatment, particularly when reconstruction using the patient’s own liver tissue is unfeasible [125]. Although the procedure can be technically demanding, particularly for IVC reconstruction, very favorable outcomes can be achieved, with low to no recurrence of AE infection [216].

An added complication to liver transplant in AE cases is the origin of the donor tissue. In the case of transplantation from a dead donor, the IVC can also be used; this is an advantage that cannot be replicated when the donated tissue is obtained from a living donor, requiring reconstruction [217]. Transplantation without IVC reconstruction can be performed [206], usually in end-stage cases in which collateral circulation is significant following IVC obstruction by the parasitic lesion [218,219]. Although collateral circulation is frequently cited as facilitating postoperative recovery, IVC clamping may not always be well tolerated [217]. Furthermore, collateral circulation in *E. multilocularis* infection is atypical and unique to this pathology compared with other etiologies [196,218]. Ning et al. described blood flow reversal caused by collateral circulation and hepatic vein obstruction [196], which can further complicate treatment options.

Transplantation can also be applied to other neighboring organs; one such case was reported by Chernyavskiy et al., who described combined heart–liver transplantation in a patient whose heart was also affected by the parasitic lesion [33].

#### 3.4.3. Antiparasitic Medication

Benzimidazole therapy (specifically albendazole) is used as an adjuvant to surgery in cases of complete resection, or as a salvage therapy in partial resections or for patients who cannot undergo surgery [127,186,220]. In some instances, benzimidazole therapy may diminish the parasitic lesion to a sufficient degree that surgery can once again become a curative option [221].

Usually, treatment with benzimidazoles requires a prolonged period of time to exert a therapeutic effect, owing to its parasitostatic nature [127]. Interruptions in treatment can be very dangerous, and can occur due to drug shortages or the appearance of adverse side-effects that can force the patient to discontinue their medication [127,222]. Furthermore, monitoring of treatment is ill-defined [127] and treatment duration varies significantly from patient to patient [222]; to mitigate this, Žideková et al. have defined a LC-MS/MS method to detect albendazole, albendazole sulfoxide and albendazole sulfone levels in plasma, which can help with avoiding adverse effects and maintaining a constant therapeutic dose in clinical settings [223].

Although benzimidazole therapy is an alternative for patients who cannot receive surgical therapy, the duration of medication required for curative effects varies from individual to individual. Imaging methods such as [^18^F][FDG] -PET/CT, which can detect differences between infected and healthy tissue, combined with the negative presence of Em18 antibodies, can offer clinicians a sign that a curative effect has been achieved [129,140,141,224].

As albendazole is a known teratogenic drug, pregnancy and postpartum care are difficult in the context of an AE patient, and the patient must be made aware of the potential risks of discontinuing treatment. Fannig et al. describe a case of a pregnant woman who required temporary discontinuation of this medication during pregnancy, but only with regular monitoring of lesion growth during the cessation of albendazole therapy [147]. This matter is discussed in further detail in Section 4.

Beyond benzimidazoles, little information is available about alternatives. For patients who cannot tolerate treatment, amphotericin B has been used with some success. However, nephrotoxicity and other adverse effects pose a similar therapeutic challenge to continued use, as is the case for albendazole [225,226].

Nitazoxanide, mefloquine, and pembromizulab have also been used experimentally, with mixed results; further studies are needed to assess whether they can serve as viable alternatives to benzimidazoles [225,226].

#### 3.4.4. Vaccine

Despite the prospect of eradicating echinococcosis by 2030 and the inclusion of a vaccine in the WHO’s plans to combat *E. granulosus*, the same cannot be said for *E. multilocularis*, for which there is no mention of a vaccine [227]. Furthermore, we have found no human studies in our search. However, promising studies on in vitro and in silico models are included in Section 4.

#### 3.4.5. Other

Beyond the aforementioned therapeutic options, several new developments have been investigated to support treatment.

Immunology may play a role in future therapeutic options. Samples from AE patients have been found to contain Natural Killer (NK) cells with a highly expressed Natural Killer cell protein Group 2-A (NKG2A); NK cells with blockage of NKG2A expression have been found to release higher concentrations of IFN-γ, TNF-α, and Granzyme B, which could increase immune response to the parasitic lesion and assist in treatment [228,229]. Macrophages, particularly those found in the liver (also known as Kupffer cells), may also hold value as potential vectors for treatment [230]; T-cell immunoglobulin and mucin domain-4 (Tim-4) in macrophages has also been found to be associated with an inadequate immune response and *E. multilocularis* immune evasion, with Tim-4 inhibition being a possible therapeutic option [231]. Another domain, Tim-3, exhibits similar properties, with high expression in AE patients [232]. A similar possibility has also been presented regarding the inhibition of the MTLN protein [233] and METTL3 [234] in M2 macrophages.

Preoperative estimates of the degree of inflammatory response, using the immune-inflammatory index and the prognostic nutritional index, can also aid in assessing postoperative prognosis [235]. The nutritional status of patients appears to be a valuable tool for patient management [236] and for predicting postoperative recovery [237].

Because *E. multilocularis* may induce T cell functional exhaustion, blocking the tyrosine-based inhibitory motif (TIGIT) domain may have clinical utility; T cells from AE patients became more active after administration of TIGIT blockers [238]. This pattern of exhaustion also extends to mucosal-associated invariant T cells, whose distribution and migration in peripheral blood may indicate their potential use as both diagnostic and therapeutic tools [239,240].

Detection of *Echinococcus* spp. cell-free DNA (cfDNA) fragments in plasma can also serve as a diagnostic tool [241,242,243]. Measurement of cell-free DNA from *E. multilocularis*, in particular, can have applications beyond diagnosis; Fan et al. [244] also propose that monitoring cell-free DNA during treatment can inform clinicians about lesion size, treatment efficacy, and the risk of recurrence after treatment cessation.

Prognostic and evolution-of-disease markers remain scarce in AE. Joliat et al. evaluated the predictive value of PD-L1 expression in patients who underwent surgery for AE; their results suggested that PD-L1 overexpression was associated with a higher recurrence risk [245]. Ke et al. focused on platelet-derived growth factor-BB (PDGF-BB) as a potential biomarker to assess the metabolic activity of AE lesions; their results suggest that the serum levels of the protein are indicative of lesion metabolic activity [246]. Other potential biomarkers include cytokeratins and pro-apoptotic proteins, such as caspase-3 and Bax [247], as well as the eosinophilic cationic protein; however, further studies are needed to confirm these results [248].

As AE is very similar to oncological disease in both pathology and severity, the psychological aspect of the patient’s health must also be considered, with the psychological burden of the disease and its progression having an important effect on the patient’s quality of life [249]. Misdiagnosis is also an essential element that can further impact the patient’s trust in the medical system and their compliance with treatment [157].

## 4. Discussion

Understanding what drives the spread of alveolar echinococcosis has been identified as understated. Autochthonous strain confirmation [42,104,105,106,107,108] confirms that control has not yet been achieved. Furthermore, in terms of prevention, nonspecific methods have yet to be fully confirmed as effective, and more specific interventions, such as vaccines, have not been fruitful as yet. Nonetheless, despite progress toward the targets set by WHO [250], the medical world has steadily integrated AE into practice, thereby slowing the rate of misdiagnosis.

Another aspect that has to be properly assessed is the economic burden of AE.

It is critical to understand that the state in which AE is present in today’s literature has greatly improved in comparison to the period prior to 2021. New avenues of research are formulated regularly, and while some objectives seem out of reach, prevention and control seem to be the next natural step in places where the epidemiological context has been defined.

### 4.1. Avenues of Research

Despite the aforementioned lack of data on human vaccine development, promising avenues of research exist. In murine models of *E. multilocularis*, antigenic targets for vaccine development have been identified, including the LTB-EMY162 recombinant immunogen [251] and EmTSP-3 and EmTIP [252].

Given the similarities between *E. granulosus* and *E. multilocularis*, using scolicidal agents during surgery to reduce the risk of protoscoleces being released into the circulation is a common practice. However, scolicidal agents are usually used only in *E. granulosus* infections. Among these, hypertonic saline is most commonly used due to its availability, though its use can often lead to severe natremia, particularly in children [253].

In one case report, a patient associated albendazole therapy with the consumption of Maca plant extract (*Lepidium meyenii*), with the disappearance of the parasitic lesion occurring 42 months post-diagnosis; further research noted that the Maca extract had no parasiticidal effects, though it did inhibit in vitro albendazole activity [254].

In cerebral AE, increases in TNF-α and VEGF-A, as well as increased microvessel density in the perilesional brain, predict perilesional brain edema [255].

Plasma concentrations of IL-27 and IL-22 were higher in patients with AE than in CE, although the difference was not statistically significant. While interleukins may have prognostic value in *E. granulosus*, their predictive value in *E. multilocularis* remains less clear [256].

Regarding the discussion of albendazole use during pregnancy, the WHO has issued a recommendation for its use in certain parasitic infections as a single dose administered after the first trimester [257], though, notably, this does not include echinococcosis, despite albendazole being the first-choice treatment for AE [258]. It is unclear whether a single dose of albendazole would be beneficial in managing a growing parasitic lesion in a pregnant patient suffering from AE.

### 4.2. Limitations of the Study

Our study is limited by intrinsic issues in its data collection methods. First, we excluded articles published in languages other than English. As we do not wish to misinterpret findings due to translation errors, we decided to exclude non-English articles altogether. However, doing so limits the scope of our study. Secondly, because published data on *E. multilocularis* from previous decades are substantial, we have limited our survey to articles published since 2020. This offers the advantage of presenting only novel findings; however, it also limits our ability to compare these findings with earlier studies. Thirdly, because all studies, regardless of spatial or temporal scope, were included, the data are heterogeneous. To this end, data were presented in a manner in which the reader could refer to relevant studies, rather than drawing a definite conclusion where one could not be drawn. Nevertheless, a limitation of the present study is the inability to draw definitive conclusions, particularly regarding epidemiology, specifically the actual spread of the disease. In this context, it is relevant to note that subpopulations within studies also showed heterogeneous data [27,79]. Thus, data synthesis could not be conducted objectively in a manner consistent with the body of knowledge; that is, one could not infer results for the general population. Finally, despite the author’s thorough search, there remains an inherent risk of bias due to the potential for unidentified works in the available literature (e.g., in other databases or mismanaged articles).

## 5. Conclusions

Diagnosis and staging of alveolar echinococcosis (AE) rely on imaging modalities such as ultrasound (US), magnetic resonance imaging (MRI), and computed tomography (CT), as well as serological testing. CT, MRI, and US are typically combined to provide a comprehensive assessment of lesions. Imaging is essential for diagnosing *Echinococcus multilocularis*. However, delays in differential diagnosis often result in most patients being identified at advanced stages of disease.

Serology can confirm the diagnosis and monitor disease progression. Serological testing supports imaging findings and, over time, helps differentiate AE from oncological pathologies. *Echinococcus multilocularis*-specific IgE levels help monitor disease progression, while total IgE levels remain stable over time.

Histopathological examination of biopsy or resection specimens can confirm a diagnosis when imaging or serology is inconclusive. This is especially valuable in cases initially suspected of oncological pathology.

Metagenomic next-generation sequencing (mNGS) can detect *E. multilocularis* DNA in samples such as abdominal lesions, cerebrospinal fluid in cerebral AE, and lung-puncture specimens.

Surgery remains the primary treatment for AE, consistent with previous findings.

Benzimidazole therapy is used as an adjuvant after complete resection or as salvage therapy when resection is partial or when surgery is not feasible. In some cases, benzimidazole therapy may reduce the lesion enough to make surgery curative.

Effective patient care requires interdisciplinary management, ideally delivered within specialized AE centers. The development of specialized AE centers is recommended. Collaboration between veterinary and human specialists is essential.

Although advances have been made in the diagnosis, management, and treatment of alveolar echinococcosis, challenges remain. Misdiagnosis, delayed diagnosis, reliance on parasitostatic-only therapy for patients who cannot undergo curative surgery, and the lack of vaccines for human patients are all areas that can be improved to enhance quality of life and treatment for patients with *E. multilocularis* infection in the 2020s.

The One Health concept deserves emphasis at every opportunity. Collaboration among specialists from different fields is also essential for studies of *E. multilocularis* infection.

## Data Availability

On request, the authors could provide the data associated with the article for further use.

## Supplementary Materials

The following supporting information can be downloaded at https://www.mdpi.com/article/10.3390/pathogens15020132/s1, Table S1: Methodology—list of articles included in systematic review; Table S2: Methodology—number of articles found based on search terms; Table S3: PRISMA 2020 checklist.
